# Integrated transcriptome and metabolome reveal *Mycoplasma hyopneumoniae* -induced lipid accumulation, inflammation, oxidative stress, and energy imbalance in the liver of pigs

**DOI:** 10.1080/21505594.2026.2679238

**Published:** 2026-06-03

**Authors:** Xiaoshu Xue, Yu Bu, Chenlong Yu, Junrong Li, Jianwei Yang, Ming Gao, Peihuan Wang, Yuan Gan, Weijing Zhang, Shuhao Fan, Ren Zhou, Zongjun Yin, Xianrui Zheng

**Affiliations:** a College of Animal Science and Technology, Anhui Agricultural University, Hefei, China; b Key Laboratory for Veterinary Bio-Product Engineering, Ministry of Agriculture and Rural Affairs, Instit Ute of Veterinary Medicine, Jiangsu Academy of Agricultural Sciences, Nanjing, China

**Keywords:** Mycoplasma hyopneumoniae, liver, inflammation, oxidative stress, energy metabolism

## Abstract

*Mycoplasma hyopneumoniae* (Mhp), the causative agent of swine enzootic pneumonia, has been linked to hepatic dysfunction, however, the underlying metabolic mechanisms remain unclear. We established an Mhp infection model and investigated hepatic responses by integrating transcriptomic and metabolomic analyses. Liver dysfunction was evaluated histologically via H&E staining and serum biochemistry. Mhp infection caused hepatic injury characterized by elevated serum aspartate aminotransferase (AST), triglyceride (TG) accumulation, increased non-esterified fatty acids (NEFAs), and neutrophil infiltration. A total of 3,241 differentially expressed genes and 685 metabolites were identified and were enriched in PPAR signaling, inflammatory, and energy metabolism pathways. Genes related to fatty acid oxidation, antioxidation, and energy metabolism were downregulated, whereas those associated with TG synthesis and inflammation were upregulated. Reduced cyclic adenosine monophosphate (cAMP) levels, decreased superoxide dismutase (SOD) activity, and elevated hydrogen peroxide (H_2_O_2_) and malondialdehyde (MDA) indicated oxidative stress and impaired energy production. These findings provide mechanistic insights into Mhp-induced hepatic metabolic injury and potential therapeutic targets.

## Introduction

*Mycoplasma hyopneumoniae* (Mhp), the primary etiological agent of swine enzootic pneumonia (SEP), is a major pathogen that causes substantial economic losses in the global swine industry [1]. Although Mhp infection mainly affects the respiratory system of pigs, its relationship with liver diseases has not been well established. Nevertheless, several studies have indicated that Mhp can alter serum metabolite profiles, especially alpha-amino butyric acid (AABA). The increase in AABA is believed to be caused by excessive protein catabolism and liver dysfunction [2]. Strains of Mhp were isolated from the livers of infected pigs [3]. Histological examination of liver tissues further indicated that Mhp infection caused liver necrosis and liver cell degeneration [4]. In reports of human infection, a notable proportion (approximately 9–30%) of patients with *Mycoplasma pneumonia* exhibit signs of hepatic dysfunction, typically manifested as elevated serum levels of liver enzymes, particularly aspartate aminotransferase (AST) [5,6]. These findings suggest a potential systemic dimension of Mhp infection, possibly involving hepatic metabolic disturbances. However, the direct effects of Mhp infection on liver function and the underlying molecular mechanisms remain insufficiently investigated and poorly understood.

The liver is a central metabolic organ responsible for vital physiological processes, including lipid metabolism, energy homeostasis, and detoxification [7]. Disruption of hepatic lipid metabolism, which frequently results in excessive lipid accumulation (hepatic steatosis), represents a pivotal event in numerous metabolic disorders [8]. The peroxisome proliferator-activated receptor alpha (PPARA) pathway serves as a key regulator of hepatic fatty acid β-oxidation [9]. Impairment of this pathway can reduce the catabolism of non-esterified fatty acids (NEFAs) and triglycerides (TGs), thereby promoting hepatic steatosis [10]. Furthermore, lipid accumulation can trigger hepatic inflammation and oxidative stress – interconnected processes that disrupt metabolic homeostasis and energy production, thereby creating a self-perpetuating cycle of metabolic dysfunction [11]. Given the reported associations between pneumonia and hepatic injury, and the liver’s central role in metabolism, it was hypothesized that Mhp infection induces systemic effects that disrupt hepatic metabolic processes. It was further proposed that Mhp infection causes transcriptional reprogramming in the liver, suppressing fatty acid oxidation and promoting lipid accumulation, which subsequently initiates inflammatory responses and oxidative stress, ultimately resulting in energy metabolism imbalance.

To test this hypothesis, a porcine model of Mhp infection was established, and a comprehensive multi-omics analysis integrating transcriptomics and metabolomics was performed. The objective of this study was to elucidate the effects of Mhp infection on hepatic fatty acid and triglyceride metabolism, inflammatory responses, oxidative stress, and energy metabolism. The findings from this study are expected to provide novel insights into the systemic metabolic consequences of Mhp infection and to establish a foundation for developing strategies that mitigate its broader impact on swine health and productivity.

## Materials and methods

### Animal experimental design

Twenty 7-week-old Landrace piglets were purchased from Anhui Antai Breeding Pig Co., Ltd. Clinical examination confirmed the absence of cardiopulmonary disorders or respiratory symptoms. None of the piglets had received any prior medication or vaccination against Mhp. Prior to inoculation, serum samples were tested for Mhp-specific antibodies, and nasal swabs collected from all animals were analyzed by nested PCR to confirm the absence of Mhp [12]. Furthermore, serological testing indicated that all piglets were negative for Classical Swine Fever Virus (CSFV), Porcine Reproductive and Respiratory Syndrome Virus (PRRSV), Porcine Circovirus Type 2 (PCV2), and Pseudorabies Virus (PRV). The piglets were housed in a dedicated animal facility with ad libitum access to water and received a standardized antibiotic-free diet throughout the study. All in vivo Mhp infection experiments were conducted in accordance with the National Guidelines for the Management and Use of Laboratory Animals (CNAS-CL06: 2018). All animal experiments were conducted in compliance with standard animal welfare guidelines and were approved by the Animal Ethics Committee of Anhui Agricultural University (AHAUXMSQ2025042).

The piglets were randomly divided into two groups. The negative control group (CON, *n* = 10) received 5 mL of sterile PBS per pig via intratracheal inoculation. The experimental group (Mhp, *n* = 10) was inoculated intratracheally with the virulent Mhp Js strain (5 × 10^8^ CCU/mL, cultured in KM2 medium) at a dose of 10 mL per animal. All pigs were observed daily for 15 minutes during the same time period to monitor the onset of respiratory symptoms. Behavioral changes including appetite, respiratory patterns, and coughing were also recorded [13].

### Sample collection

After performing artificial inoculation with Mhp, the macroscopic lesions in the lungs usually reach their most severe stage between the 3rd and 4th weeks (21dpi to 28dpi) [14,15]. Therefore, the experimental endpoint was set at 28 days post-infection. Blood samples were obtained from the jugular vein prior to morning feeding at 28dpi [16]. Samples were centrifuged at 3000 rpm for 15 minutes to separate serum and plasma, which were stored in liquid nitrogen. Piglets in the Mhp-inoculated and control groups were euthanized via intravenous injection with a lethal dose of sodium pentobarbital (Sinopharm) at 28dpi. Each lung was evaluated for the presence of typical lesions, such as “EP-like” consolidation located in the apical, intermediate, accessory and cranial portions of the diaphragmatic lobes [17]. Immediately after euthanasia, necropsy was performed, liver samples were harvested from the left lobes of pigs in both the CON and Mhp groups within 30 minutes. Each liver sample was divided for preservation: one part was flash-frozen in liquid nitrogen for metabolic and gene expression profiling, and the other was immersion-fixed in 4% paraformaldehyde for paraffin embedding and hematoxylin-eosin staining.

In addition, the BALF of each pig was collected after necropy as well, centrifuged and stored at − 70 °C until use. Lungs from the two infected groups (samples were taken from lesion margins) and from the two control groups (samples were taken routinely from the intermediate lobes of the right lung) were harvested from the pigs after death (after euthanasia at the end of the trial) and were collected for further pathological observation.

### Mhp strain

The virulent Mhp strain Js was kindly provided by the Jiangsu Academy of Agricultural Sciences. Mhp strain Js is a pathogenic strain that can trigger the typical endemic pneumonia characteristics of traditional pigs [16], and the virulence of which was assessed in our previous report [18]. Its titer was determined using the color change unit (CCU) assay in KM2 cell-free liquid medium – a modified Friis formulation containing 20% (v/v) porcine serum – and was additionally verified by TaqMan quantitative PCR [19,20].

DNA of Mhp was extracted from 0.2 mL of BALF of each piglet using a DNA Extraction Kit (Axygen, Cat.No: AP-MN-BF-VNA-250, New York, USA). Extracted DNA was quantified by quantitative real-time PCR using the TaqMan system, and primers and TaqMan-TAMRA probes were designed based on the conserved sequence of the P97 gene of Mhp (The primer sequences of the P97 gene are shown in Table S1). In addition, a standard curve was generated using a 10-fold diluted standard plasmid, PMD-T-P97, as a template before using a QuantStudio R5 Real-Time PCR System to determine the DNA copies as described previously [19,20]. The samples were run in triplicate biological replicates.

### Serological tests

Serum samples were collected from all piglets at 0 and 28 dpi and tested for Mhp-specific antibodies using a commercially available ELISA kit (99–06733, IDEXX, Liebefeld-bern, Switzerland) according to the manufacturer’s instructions [21]. Based on the test results, the s/p value (sample mean/positive control mean) of the serum Mhp antibody was used as the criterion for determining whether the antibody was positive or negative. A s/p value less than 0.3 was defined as a negative Mhp antibody, while a s/p value greater than 0.4 was defined as a positive Mhp antibody.

### Histological studies

Histological analysis of lung and liver slices was performed with and without Mhp infection. The lung and liver samples was immersed in 4% paraformaldehyde at 4°C for 24 h. After dehydration through an alcohol gradient, clearing, and embedding in paraffin wax, the sliced sections (4 μm) were stained with hematoxylin and eosin (H&E).

### Scanning electron microscopy observation

Bronchus samples were taken from the apical lobe of each lung for frozen sectioning, and the ultrastructure of the bronchial surfaces was examined by scanning electron microscopy (EVO-LS10, Zeiss, Germany).

### Analysis of biochemical indicators and oxidative stress indices

Serum alanine aminotransferase (ALT), AST, and alkaline phosphatase (ALP) levels were analyzed via an automated Stratus CS system (Dade Behring, Deerfield, IL, USA). Serum and hepatic levels of NEFAs and TG were assessed with commercial assay kits from the Jiancheng Bioengineering Institute (Nanjing, China), in accordance with the manufacturer’s protocols. Similarly, hepatic oxidative stress indices, including H_2_O_2_, MDA, and SOD, were quantified via corresponding kits from the same manufacturer, following the provided guidelines.

### Analysis of inflammatory indicators

Liver tissues were collected, and cytokine levels, including IL-1β, IL-6, and TNFα, were quantified using commercially available ELISA kits (IL1β: ab100754; IL6: ab100755; TNFα: ab100756; all from ABCAM, USA) in accordance with the manufacturer’s instructions.

### Hepatic transcriptome analysis

Total RNA was extracted from liver tissue via TRIzol reagent (Invitrogen, USA) according to the manufacturer’s instructions. RNA quality was assessed using a NanoDrop ND-1000 spectrophotometer, ensuring A260/230 and A260/280 ratios between 1.80 and 2.10. Ten RNA samples (5 per group) were randomly selected for cDNA library construction. Briefly, mRNA was purified from total RNA using the magnetic bead method, then fragmented and reversely transcribed into cDNA. The purified cDNA was added sequencing adapters and amplified using the NEBNext® Ultra™ RNA Library Preparation Kit. Fragments with appropriate length were selected to establish a cDNA library, which was sequenced on an Illumina NovaSeq 6000 (Wuhan, China). Clean reads were generated by removing the low-quality reads and aligned to the Sscrofa11.1 using HiSAT2. Gene expression was quantified using FPKM values. Differentially expressed genes (DEGs) were identified using DESeq2 software (v.1.6.3) with a false discovery rate (FDR) <0.05 and fold change (FC) >2 or <0.5. The total genes in the liver tissues were analyzed using principal component analysis (PCA) in SIMCA v.14.1 software (Umetrics, Umea, Sweden).

### Hepatic metabolome analysis

Ten liver samples (5 per group) were randomly selected for metabolome analysis. In untargeted metabolome analysis, chromatographic separation was performed under the following conditions: the column temperature was maintained at 40 °C, the flow rate was 0.2 mL/min. For positive ionization mode, mobile phase A consisted of 0.1% (v/v) formic acid in water, and mobile phase B was methanol. For negative ionization mode, mobile phase A was 6 mmol/L ammonium acetate in water (adjusted the pH to 9.0 with ammonium hydroxide), and mobile phase B was methanol. SIEVE software (Thermo Fisher Scientific) was used to analyze the mass spectrum data. The online database Human Metabolome Database (HMDB; http://www.hmdb.ca) and KEGG were used to identify metabolites by matching the molecular mass data. Commercial reference standards were utilized to validate and confirm the hepatic metabolites with high confidence by comparison of their MS/MS spectra and retention time. The *P* values of the metabolites were calculated using non-parametric method in IBM Statistics SPSS 26, which were corrected for multiple testing using the Benjamini – Hochberg method to obtain the FDR. Overall metabolic profile in the liver tissues were analyzed using PCA in SIMCA v.14.1 software (Umetrics, Umea, Sweden). Finally, differential metabolites were selected according to the criteria of variable importance in projection (VIP) >1, FDR < 0.05, and FC > 1.5 or <0.67. Differential metabolites were further analyzed and enriched using the MetaboAnalyst web server (http://www.metaboanalyst.ca). A permutation test with 200 iterations was applied to validate model robustness. Identified metabolites were annotated using Kyoto Encyclopedia of Genes and Genomes (KEGG) Compound database (http://www.kegg.jp/kegg/compound/), annotated metabolites were then mapped to KEGG Pathway database (http://www.kegg.jp/kegg/pathway.html).

### Real-time quantitative PCR

The mRNA expression levels of key genes were analyzed via RT‑qPCR. The primer sequences for the target genes (Table S1) were designed with Primer3 software and synthesized by General Biology Co., Ltd. (Chuzhou, China). Each 20 μL RT‑qPCR reaction contained 10 μL of SYBR Green I Master Mix, 0.8 μL of each primer, 0.4 μL of ROX Reference Dye II (60×), 2 μL of cDNA template, and 6 μL of nuclease-free water. Amplification was carried out on an ABI 7600 Real-Time PCR System (Thermo Fisher Scientific). Gene expression was normalized to that of GAPDH and calculated via the 2^^–ΔΔCt^ method.

### Statistical analysis

Prior to the experiment, a power analysis was conducted to determine the minimal sample size required. On the basis of an independent-samples t-test, a sample size of 10 pigs per group was sufficient to detect an effect size of 1.7 standard deviations (SDs) in key outcome measures related to Mhp infection, with a statistical power of 95% and a type I error rate of 5%. The RT-qPCR data, oxidative stress indicators, and inflammatory indicators were analyzed via two-tailed t-test for comparisons between two groups. GraphPad Prism v.9.0 software was used for graphic drawing, and all data are expressed as the mean ± SEM. *p* < 0.05 was considered as statistically significant.

## Results

### Mhp infection causes liver inflammation and dysfunction

**Figure 1.**
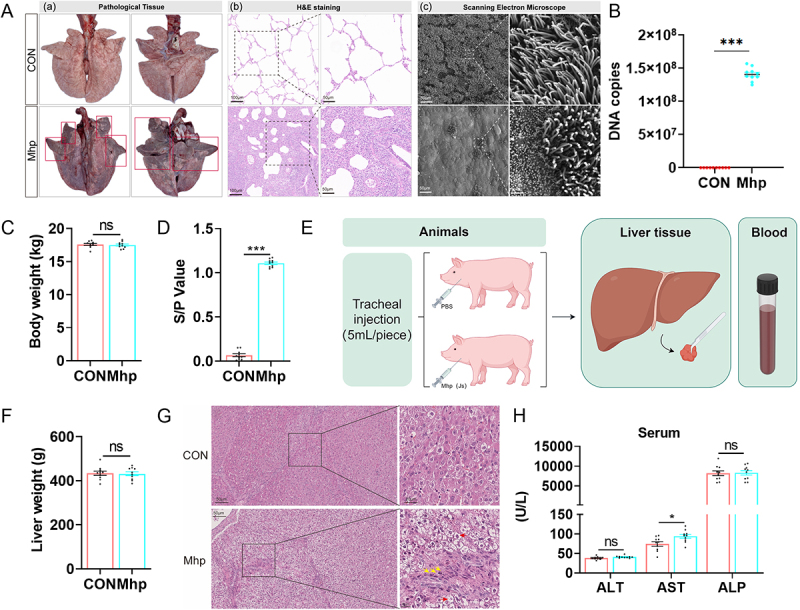
Mhp infection leads to liver inflammation. **A** Mhp causes severe respiratory infections. **(a)** Gross lesions evaluation of lung samples at 28 dpi after necropsy. Whole lung samples connected to the trachea of each piglet were imaged after the animals were sacrificed. **(b)** Histopathological manifestations (H&E) of lung samples in the control group (CON) and the Mhp infection group (Mhp). Images with magnifications of 10× and 20× are respectively displayed. **(c)** Scanning electron microscopy observation observation of bronchi ciliary structure. Images with magnifications of 1× and 10× are respectively displayed. **B** quantitative PCR assay of Mhp from the BALF of all pigs. The vertical axis shows the DNA copy number of the Mhp P97 gene. **C** comparison of pig body weight between the control group (CON) and the Mhp infection group (Mhp). **D** ELISA detection of serum Mhp in the control group (CON) and the Mhp infection group (Mhp) (*n* = 10). The S/P value was detected by commercial ELISA kits as follows: (sample outer diameter_650_- negative control OD_650_)/ (positive control OD_650_- negative control OD_650_). An s/p value higher than the critical value of 0.4 is considered positive. **E** flowchart for collecting liver and blood samples after Mhp infection. **F** comparison of liver weight between the control group (CON) and the Mhp infection group (Mhp). **G** histopathological manifestations (H&E) of liver samples in the control group (CON) and the Mhp infection group (Mhp). Images with magnifications of 20× and 63× are respectively displayed. The red arrow indicates the presence of fat vacuolar degeneration in the cells, while the yellow arrow indicates neutrophil infiltration. **H** serum alt, ast and ALP contents. * indicates *p* < 0.05. *** indicates *p* < 0.01. ns indicates no significant difference.

To examine the effects of Mhp infection on hepatic metabolism, a porcine infection model was established. First, we conducted an analysis of the impact of Mhp infection on the respiratory tract (Figure 1A). During the experiment, all the piglets infected with Mhp exhibited symptoms such as labored breathing and coughing. At 28 dpi, gross lung lesions were observed in all pigs and evaluated after necropsy. The Mhp group exhibited well-demarcated lesions, and typical “EP-like” lesions with dark red-to-purple areas of consolidation were observed, which were mainly located in the apical and intermediate lobes of the left and right lungs and accessory lobes (Figure 1A(a)). Histopathological observation of lungs from Mhp strain infected showed widened alveolar space, resulting in almost no visible alveolar structures. Meanwhile, in the Mhp group, the bronchial lumen became narrower, and a large number of inflammatory cells were observed in the alveolar spaces and within the bronchial lumen (Figure 1A(b)). Scanning EM of the bronchial tissues revealed that both Bama miniature pigs and conventional pigs infected with the Mhp strain exhibited the severe destruction of ciliary function (Figure 1A(c)). We collected BALF samples from all the piglets and quantified the Mhp DNA copy number in each piglet’s body through quantitative real-time PCR. The Mhp DNA copy number in infected piglets exceeded 10^8^ (Figure 1B). No significant differences in body weights were observed between the control (CON) and Mhp-infected groups (Figures 1C). A s/p ratio exceeding 0.4 was regarded as positive for Mhp infection, whereas a ratio below 0.3 was defined as negative. ELISA detection of Mhp-specific antibodies at day 28 confirmed the successful establishment of the infection model (Figure 1D). We collected liver and blood samples from all the experimental piglets for further analysis of liver function (Figure 1E). Similarly, weighing the freshly dissected liver did not reveal any weight difference between the control group (CON) and the Mhp-infected group (Mhp) (Figure 1F). However, H&E staining revealed pronounced lipid droplet accumulation and neutrophil infiltration in the livers of Mhp-infected pigs (Figure 1G). Serum AST levels were significantly elevated in the Mhp group, whereas ALT and ALP levels did not differ significantly between groups (Figure 1H). These findings indicate that Mhp infection impaired hepatic function; however, the underlying regulatory mechanisms remain to be elucidated.

### Transcriptomic analysis revealed Mhp-induced disruption of hepatic lipid metabolism

**Figure 2.**
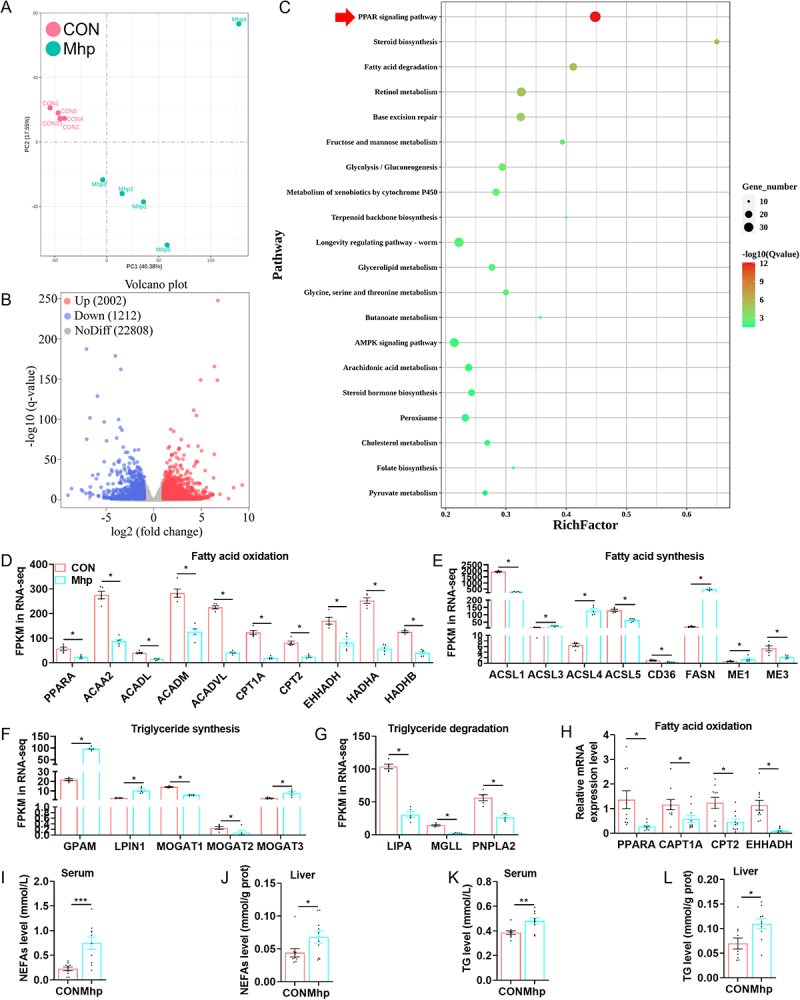
The effect of Mhp infection on the transcriptional profile of pig liver. **A** liver transcriptome PCA analysis. **B** liver transcriptome volcano plot. **C** liver transcriptome KEGG enrichment analysis. **D-G** the expression of genes related to fatty acid oxidation, fatty acid synthesis, TG synthesis, and TG degradation by transcriptome sequencing. **H** the expression of genes related to fatty acid oxidation by RT-qPCR. **I-L** serum and liver NEFAs, BHBA, TG, and TC contents. * indicates *p* < 0.05. ** indicates *p* < 0.01. ns indicated no significant difference.

PCA of the hepatic transcriptomes revealed a distinct separation between the CON and Mhp groups (Figure 2A). A total of 2,002 genes were upregulated and 1,212 were DEGs were identified between the two groups based on volcano plot analysis (Figure 2B). KEGG enrichment analysis indicated that the PPAR signaling pathway was significantly enriched among these DEGs (Figure 2C). Compared with the CON group, the Mhp-infected group showed significant downregulation of genes associated with fatty acid β-oxidation, including *PPARA, ACAA2, ACADL, ACADM, ACADVL, APT1A, CPT2, EHHADH, HADHA, and HADHB* (Figure 2D). Additionally, Mhp infection downregulated fatty acid synthesis-related genes (*ACSL1, ACSL5*, and *CD36*) while upregulating *ACSL3, ACSL4, FASN, ME1*, and *ME3* (Figure 2E). Genes involved in TG synthesis (*GPAM, LPIN1*, and *MOGAT3*) were significantly upregulated in the Mhp group, whereas *MOGAT1* and *MOGAT2* were downregulated (Figure 2F). Conversely, genes associated with TG degradation (*LIPA, MGLL*, and *PNPLA2*) were markedly downregulated (Figure 2G). RT-qPCR further confirmed the downregulation of key genes involved in fatty acid oxidation (Figure 2H). Biochemical assays revealed that Mhp infection increased NEFA and TG levels in both serum and hepatic tissue (Figures 2I–L). Collectively, these findings indicate that Mhp infection disrupted hepatic transcriptional regulation, impaired fatty acid oxidation, and promoted lipid accumulation in the liver.

### Metabolomic analysis reveal Mhp-induced disruption of hepatic lipid metabolism

**Figure 3.**
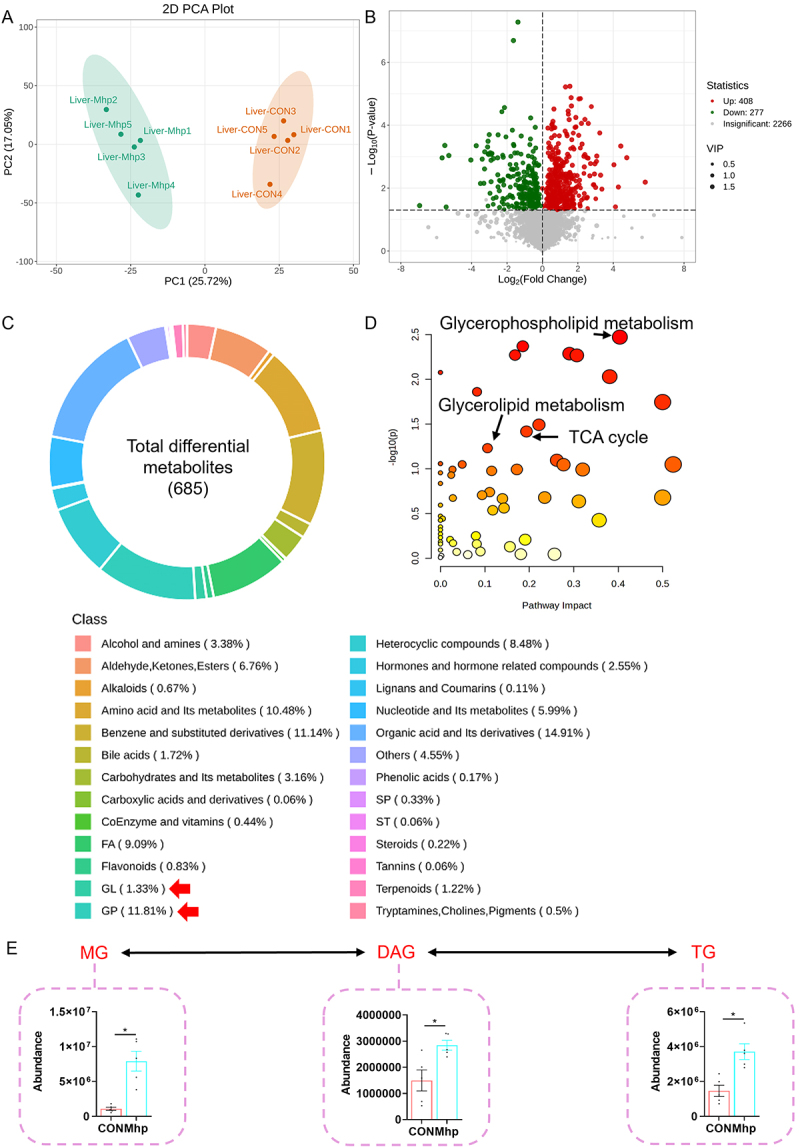
The effect of Mhp infection on the metabolic profile of pig liver. **A** liver metabolome PCA analysis. **B** liver metabolome volcano plot. **C** distribution of DEMs in the liver metabolic profile. **D** liver metabolome KEGG enrichment analysis. **E** the abundance of MG, DAG, and TG in the liver metabolism profile. FA, fatty acid; gl, glycerolipid; GP, glycerophospholipid; SP, sphingolipids; st, sterol lipid. * indicates *p* < 0.05.

Untargeted metabolomic profiling of porcine livers revealed distinct clustering between the CON and Mhp groups (Figure 3A). A total of 685 differentially expressed metabolites (DEMs) were identified, including 408 upregulated and 277 downregulated metabolites (Figure 3B). The identified metabolites belonged to diverse chemical categories, including amino acids and derivatives, fatty acids, glycerophospholipids, organic acids, and other minor classes (Figure 3C). KEGG enrichment analysis demonstrated significant enrichment of glycerophospholipid metabolism, glycerolipid metabolism, and TCA cycle pathways (Figure 3D). With respect to glycerolipid metabolism, the hepatic abundances of MG, DAG, and TG were markedly elevated following Mhp infection (Figure 3E). Moreover, 25 hepatic free fatty acids were significantly increased and 5 were decreased after infection (Table S2). Collectively, these results indicate that Mhp infection disrupted hepatic lipid metabolism and promoted lipid accumulation.

### Mhp induced hepatic oxidative stress and inflammation

**Figure 4.**
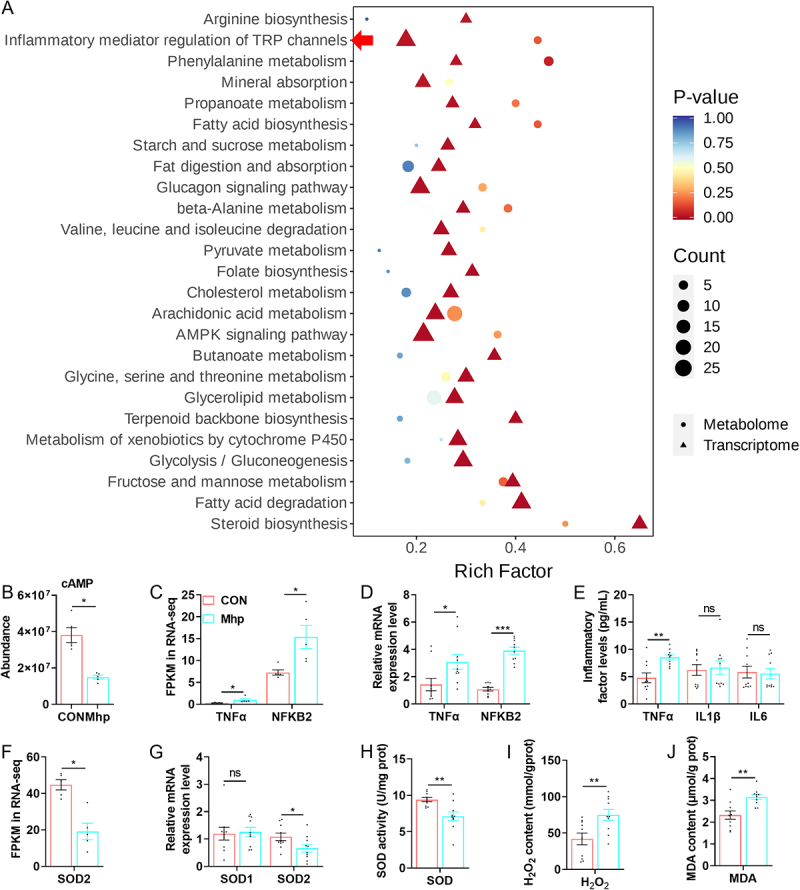
Joint analysis of transcriptional profile and metabolic profile by Mhp on the effects of liver inflammation and oxidative stress. **A** KEGG network obtained through combined enrichment of transcriptional profiles and metabolic profiles. **B** the abundance of cAMP in the liver metabolism profile. **C** the expression of genes related to inflammation by transcriptome sequencing. **D** the expression of genes related to inflammation by RT-qPCR. **E** secretion levels of inflammatory factors TNFα, IL1β, and IL6 in the liver. **F** the expression of genes related to antioxidation by transcriptome sequencing. **G** the expression of genes related to antioxidation by RT-qPCR. **H-J** the levels of sod, H_2_O_2_, and MDA in the liver. * indicates *p* < 0.05. ** indicates *p* < 0.01. *** indicates *p* < 0.001. ns indicated no significant difference.

Lipid accumulation in the liver is known to trigger inflammatory responses and oxidative stress. Integrated transcriptomic – metabolomic analysis within the KEGG framework revealed significant enrichment of differentially expressed genes and metabolites in the pathway of inflammatory mediator regulation of TRP channels (Figure 4A). Notably, the abundance of cAMP, a key inflammatory regulator, was markedly lower in the Mhp group compared than in the CON group (Figure 4B). Transcriptomic profiling revealed significant upregulation of the proinflammatory gene TNFα and the key signaling molecule NFKB2 in the Mhp group, which was further validated by RT-qPCR (Figures 4C,D). ELISA revealed that hepatic TNFα secretion was significantly elevated in the Mhp group, whereas IL-1β and IL-6 levels remained unchanged. Given the established association between hepatic inflammation and oxidative stress, related oxidative markers were also examined. Transcriptomic and RT-qPCR analyses demonstrated that SOD2 expression was significantly downregulated in the Mhp group, whereas SOD1 expression remained unchanged (Figures 4F,G). Consistently, hepatic SOD activity was significantly decreased in the Mhp group, accompanied by elevated H_2_O_2_ and MDA levels (Figures 4H–J). These findings indicate that Mhp infection induced pronounced hepatic inflammation and oxidative stress.

### Mhp induced hepatic energy metabolism imbalance

**Figure 5.**
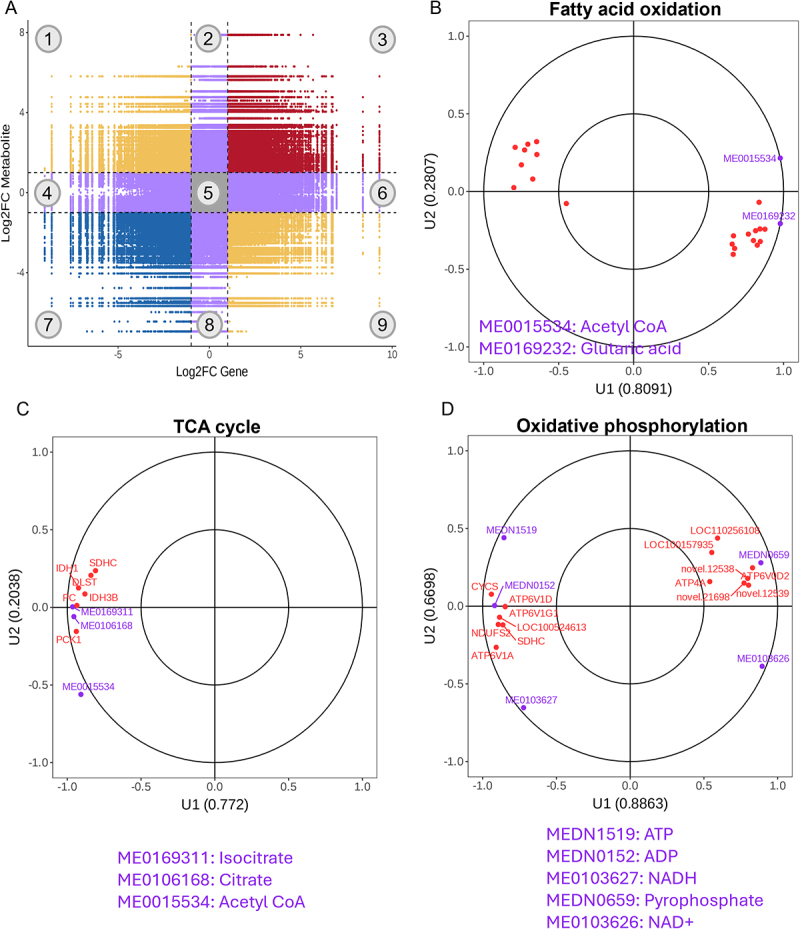
Correlation analysis of transcription profiles and metabolic profiles. **A** correlation analysis nine-quadrant chart. Within each differential group, correlations meeting the criteria of an absolute Pearson correlation coefficient greater than 0.8 and a *p*-value less than 0.05 were selected. Subsequently, a nine-quadrant diagram was used to visualize the differential Fold changes of genes and metabolites associated with these correlations. Black dashed lines were employed to sequentially partition the diagram into 1–9 quadrants from left to right and top to bottom. **B-D** canonical correlation analysis of fatty acid oxidation, TCA cycle, and oxidative phosphorylation. The horizontal and vertical axes represented the simple correlation coefficients between different omics substances and typical variables U1 and U2, respectively. Purple and red dots denoted metabolites and genes, respectively. The figure divided the plane into four quadrants using cross markers. Within each quadrant, points farther from the origin indicated stronger association with the typical variables; points closer together indicated similar association strengths. To avoid text overlap, only partial substance names were displayed.

**Figure 6.**
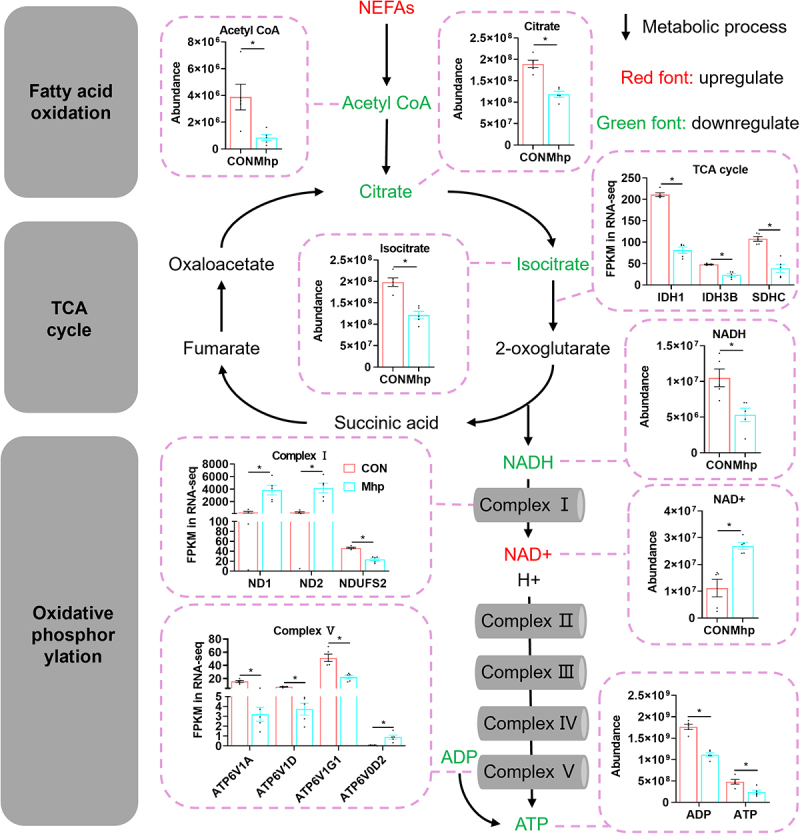
The effect of Mhp infection on liver energy metabolism. The purple box showed the abundance of metabolites (acetyl CoA, citrate, isocitrate, NADH, NAD^+^, ADP, and ATP) and the expression levels of genes associated with TCA cycle and oxidative phosphorylation. * indicates *p* < 0.05.

A nine-quadrant correlation plot was used to visualize the relationships between DEGs and DEMs. Quadrants 1 and 9 indicated opposite expression trends, quadrants 3 and 7 indicated concordant trends, while quadrants 2, 4, 6, and 8 represented non-significant correlations (Figure 5A). Fatty acid oxidation, TCA cycle, and oxidative phosphorylation pathways were enriched within quadrants 3 and 7 (Figure 5A). Canonical correlation analysis further confirmed that differentially abundant metabolites within these pathways were positively correlated with corresponding DEGs (Figures 5B–D). Compared with the CON group, hepatic acetyl-CoA, citrate, isocitrate, NADH, ADP, and ATP levels were significantly lower in the Mhp group, whereas NAD^+^ levels were significantly elevated (Figure 6). Impaired fatty acid oxidation, as evidenced by downregulation of related genes (Figure 2D), resulted in reduced acetyl-CoA production from NEFAs. Although complex I genes *ND1* and *ND2* were upregulated, significant downregulation of *NDUFS2* contributed to increased NAD^+^ generation from NADH. Simultaneously, downregulation of complex V genes (*ATP6V1A*, *ATP6V1D*, and *ATP6V1G1*) and upregulation of *ATP6V0D2* collectively led to reduced ATP synthesis from ADP. Collectively, these findings demonstrate that Mhp infection suppressed the TCA cycle and oxidative phosphorylation in the liver, leading to reduced ATP production.

## Discussion

Mhp is a major pathogen that causes significant economic losses to the global swine industry. This study investigated the association between Mhp infection and hepatic metabolic dysfunction in pigs, and further elucidated the underlying mechanisms involved. Although Mhp infections mainly affect the respiratory system, they may also induce atypical systemic manifestations. Similar clinical manifestations have been reported in humans. Some researchers have reported that the probability of liver damage after *Mycoplasma pneumonia* infection ranges from 9% to 30% [5,22,23]. Furthermore, regarding the findings from hematological research, it was discovered that after *Mycoplasma pneumonia* infection, there is an increase in neutrophils in the liver, accompanied by an elevation in AST levels [5]. These results provide a reference for understanding the mechanism of systemic transmission of Mhp infection leading to liver dysfunction. In this study, we observed that Mhp infection increased serum AST levels and elevated concentrations of NEFAs and TG in both serum and liver tissue, accompanied by lipid droplet accumulation and neutrophil infiltration in the liver. We concluded that Mhp infection induced metabolic injury in the liver, although the precise regulatory mechanisms require further investigation.

The PPARA signaling pathway plays a crucial role in regulating hepatic metabolism, particularly fatty acid oxidation [24]. It activates the transcription of key enzymes involved in fatty acid oxidation, such as *ACOX1*, *OCTN2*, *CPT1A*, and *CPT2. CPT1A* and *CPT2* facilitated the transfer of long-chain fatty acids into the mitochondrial matrix, where they undergo β-oxidation to produce energy [25,26]. By promoting the expression of these enzymes, PPARA helped ensure efficient mitochondrial fatty acid oxidation, thereby reducing hepatic accumulation of fatty acids and triglycerides [27,28]. In this study, transcriptomic profiling revealed that Mhp infection likely induced hepatic lipid accumulation primarily through the PPAR signaling pathway, fatty acid degradation, and fatty acid biosynthesis. Both transcription profile and RT-qPCR results demonstrated the downregulation of key genes involved in fatty acid oxidation after Mhp infection, including *PPARA*, *CPT1A*, and *CPT2*. Further, after Mhp infection, the expression of genes related to triglyceride synthesis in the liver is upregulated, while the expression of genes related to triglyceride degradation is downregulated. Metabolomic analysis further showed significant increases in hepatic levels of MG, DG, and TG after infection, supporting the conclusion that Mhp promotes lipid accumulation. Thus, Mhp infection suppressed the PPARA pathway, impairing fatty acid oxidation and triglyceride synthesis and degradation, ultimately leading to metabolic dysfunction.

It is well established that lipid accumulation can trigger hepatic inflammation. cAMP, a critical second messenger, is known to regulate inflammatory responses [29]. Moreover, NF-κB-dependent gene transcription was often suppressed by elevated cAMP levels [30]. Interestingly, the expression of genes such as IL1β, IL6, and TNFα—primarily driven by the transcription factor NF-κB – was inhibited by increased cAMP [31]. This suggested that elevating cAMP may suppress inflammatory cytokine expression by inhibiting NF-κB. In this study, metabolomic analysis revealed a significant decrease in hepatic cAMP abundance following Mhp infection. Concurrently, gene expression levels of *NFKB2* and *TNFα* were significantly elevated, along with increased secreted TNFα protein levels, collectively promoting hepatic inflammation. Furthermore, inflammation is closely linked to oxidative stress. Previous studies have shown that H_2_O_2_ could activate the NF-κB pathway, upregulating inflammatory cytokines such as IL1β, IL6, and TNFα, which in turn further activated NF-κB and exacerbated oxidative stress [32,33]. Additionally, high levels of NEFAs could induce oxidative stress in hepatocytes, characterized by elevated oxidative markers (H_2_O_2_ and MDA) and reduced antioxidant capacity (e.g. SOD activity) [34]. Consistent with this, we observed increased hepatic H_2_O_2_ and MDA levels and decreased SOD activity after Mhp infection, indicating oxidative damage. Therefore, Mhp infection enhanced liver inflammation and oxidative stress.

As the central organ for energy metabolism, the liver supplies the body with ATP [35]. However, lipid metabolic disorders, inflammation, and oxidative stress could disrupt hepatic energy metabolism [35]. Specifically, dysregulated lipid metabolism led to elevated NEFAs, which suppressed the expression of genes and proteins related to oxidative phosphorylation complexes I – V [36]. Moreover, treatment with IL-1β and TNFα had been shown to reduce the expression of genes involved in the TCA cycle, basal respiration, and ATP production in human primary chondrocytes, leading to energy imbalance [37]. In this study, Mhp infection impaired hepatic fatty acid oxidation, resulting in reduced acetyl-CoA production. It also downregulated genes involved in the TCA cycle, leading to decreased levels of citrate, isocitrate, and NADH. Furthermore, infection significantly downregulated the expression of genes encoding complexes I and V, ultimately reducing ADP and ATP production and suppressing hepatic energy generation.

In conclusion, our research has demonstrated that Mhp infection leads to liver metabolic damage by disrupting lipid metabolism, inducing inflammation and oxidative stress. These findings provide a mechanistic foundation for future investigations into Mhp-induced hepatic metabolic injury and for the development of targeted therapeutic interventions.

## Supplementary Material

Author Checklist.pdf

## Data Availability

Raw data generated in this study were deposited in the Sequence Read Archive Database at https://dataview.ncbi.nlm.nih.gov/object/PRJNA1331453?reviewer=oohfco47kn4h4vh6aqnsm0tu08 [38] under accession number PRJNA1331453. The datas that support the findings of this study are openly available in “figshare” at https://doi.org/10.6084/m9.figshare.30478328 [39].

## Abbreviations

Mhp: *Mycoplasma hyopneumoniae*
DEG: Differentially expressed gene
DEM: Differentially expressed metabolite
AST: Aspartate aminotransferase
ALT: Alanine aminotransferase
ALP: Alkaline phosphatase
TG: Triglyceride
NEFA: Non-esterified fatty acid
SOD: Superoxide dismutase
cAMP: cyclic adenosine monophosphate
H_2_O_2_: Hydrogen peroxide
MDA: Malondialdehyde

## Declarations

No human participant was included in our study. The detailed protocols of animal experiments are presented in Materials and Methods. Animal procedures were approved and consented by the Experimental animal Welfare and Ethics Committee of Anhui Agricultural University.

## References

[1] Maes D, Sibila M, Kuhnert P, et al. Update on Mycoplasma hyopneumoniae infections in pigs: knowledge gaps for improved disease control. Transbound Emerg Dis. 2018;65:110–14. doi: 10.1111/tbed.12677 28834294

[2] Surendran Nair M, Yao D, Chen C, et al. Serum metabolite markers of early Mycoplasma hyopneumoniae infection in pigs. Vet Res. 2019;50(1):98. doi: 10.1186/s13567-019-0715-2 31771624 PMC6878661

[3] Marois C, Le Carrou J, Kobisch M, et al. Isolation of Mycoplasma hyopneumoniae from different sampling sites in experimentally infected and contact SPF piglets. Vet Microbiol. 2006;120(1–2):96–104. doi: 10.1016/j.vetmic.2006.10.015 17116374

[4] Kolych V, Hudz N. PATHOHISTOLOGICAL changes in PIGS with MYCOPLASMOSIS. Ukr J Vet Sci. 2021;12(4). doi: 10.31548/ujvs2021.04.009

[5] Kim KW, Sung JJ, Tchah H, et al. Hepatitis associated with Mycoplasma pneumoniae infection in Korean children: a prospective study. Korean J Pediatr. 2015;58(6):211. doi: 10.3345/kjp.2015.58.6.211 26213549 PMC4510354

[6] Izumikawa K. Clinical features of severe or fatal Mycoplasma pneumoniae pneumonia. Front Microbiol. 2016;7:800. doi: 10.3389/fmicb.2016.00800 27313568 PMC4888638

[7] Yoon H, Shaw JL, Haigis MC, et al. Lipid metabolism in sickness and in health: emerging regulators of lipotoxicity. Mol Cell. 2021;81(18):3708–3730. doi: 10.1016/j.molcel.2021.08.027 34547235 PMC8620413

[8] Geng Y, Faber KN, de Meijer VE, et al. How does hepatic lipid accumulation lead to lipotoxicity in non-alcoholic fatty liver disease? Hepatol Int. 2021;15(1):21–35. doi: 10.1007/s12072-020-10121-2 33548031 PMC7886759

[9] Gong L, Wei F, Gonzalez FJ, et al. Hepatic fibrosis: targeting peroxisome proliferator-activated receptor alpha from mechanism to medicines. Hepatology. 2023;78(5):1625–1653. doi: 10.1097/HEP.0000000000000182 36626642 PMC10681123

[10] Fabbrini E, Magkos F. Hepatic steatosis as a marker of metabolic dysfunction. Nutrients. 2015;7(6):4995–5019. doi: 10.3390/nu7064995 26102213 PMC4488828

[11] Rinaldi L, Pafundi PC, Galiero R, et al. Mechanisms of non-alcoholic fatty liver disease in the metabolic syndrome. A narrative review. Antioxidants. 2021;10(2):270. doi: 10.3390/antiox10020270 33578702 PMC7916383

[12] Lu X, Feng Z, Liu M, et al. Establishment of a nested PCR assay for detection of Mycoplasma hyopneumoniae. Jiangsu J Agric Sci. 2010;26(1):91–95.

[13] Maes D, Verdonck M, Deluyker H, et al. Enzootic pneumonia in pigs. Vet Q. 1996;18(3):104–109. doi: 10.1080/01652176.1996.9694628 8903144

[14] Villarreal I, Maes D, Vranckx K, et al. Effect of vaccination of pigs against experimental infection with high and low virulence Mycoplasma hyopneumoniae strains. Vaccine. 2011;29(9):1731–1735. doi: 10.1016/j.vaccine.2011.01.002 21237277

[15] Kobisch M, Friis NF. Swine mycoplasmoses. Rev Sci Tech OIE. 1996;15(4):1569–1605. doi: 10.20506/rst.15.4.983 9190026

[16] Gan Y, Xie X, Zhang L, et al. Establishment of a model of Mycoplasma hyopneumoniae infection using bama miniature pigs. Food Prod Process Nutr. 2020;2(1):19. doi: 10.1186/s43014-020-00034-w

[17] Garcia-Morante B, Segalés J, Fraile L, et al. Assessment of Mycoplasma hyopneumoniae-induced pneumonia using different lung lesion scoring systems: a comparative review. J Comp Pathol. 2016;154(2–3):125–134. doi: 10.1016/j.jcpa.2015.11.003 26774274

[18] Xiong Q, Wei Y, Feng Z, et al. Protective efficacy of a live attenuated Mycoplasma hyopneumoniae vaccine with an ISCOM-matrix adjuvant in pigs. Vet J. 2013;199(2):268–274. doi: 10.1016/j.tvjl.2013.11.001 24314715

[19] Furr P, Taylor-Robinson D. Factors influencing the ability of different mycoplasmas to colonize the genital tract of hormone-treated female mice. Int J Exp Pathol. 1993;74(1):97. 8471540 PMC2002218

[20] Yuzi W, Ishag HZ, Lizhong H, et al. Establishment and application of a real-time, duplex PCR method for simultaneous detection of Mycoplasma hyopneumoniae and Mycoplasma hyorhinis. KAFKAS ÜNIVERSITESI VETERINER FAKÜLTESI DERGISI. 2019;25(3). doi: 10.9775/kvfd.2018.21137

[21] Bai Y, Gan Y, Hua L-Z, et al. Application of a sIgA-ELISA method for differentiation of Mycoplasma hyopneumoniae infected from vaccinated pigs. Vet Microbiol. 2018;223:86–92. doi: 10.1016/j.vetmic.2018.07.023 30173757

[22] Watanabe Y, Kanayama H, Kato K, et al. Liver disorders in patients with Mycoplasma pneumoniae pneumonia. Jpn J Thorac Dis. 1991;29(6):693–697. 1895585

[23] Lee JT, Kim HS, Tchah H. Hepatitis complicated with Mycoplasma pneumoniae infection. Korean J Pediatr Gastro Nutr. 2001;4(2):207–212. doi: 10.5223/kjpgn.2001.4.2.207

[24] Gonzalez FJ, Xia Y. Adipose triglyceride lipase as a target for treatment of metabolic dysfunction-associated steatohepatitis: the role of hepatic and intestinal PPARα. J Hepatol. 2025;82(4):556–559. doi: 10.1016/j.jhep.2024.10.046 39542137

[25] Wang S, Kong F, Zhang X, et al. Disruption of hindgut microbiome homeostasis promotes postpartum energy metabolism disorders in dairy ruminants by inhibiting acetate-mediated hepatic AMPK-PPARA axis. Microbiome. 2025;13(1):167. doi: 10.1186/s40168-025-02150-6 40671160 PMC12265336

[26] Lee J, Choi J, Scafidi S, et al. Hepatic fatty acid oxidation restrains systemic catabolism during starvation. Cell Rep. 2016;16(1):201–212. doi: 10.1016/j.celrep.2016.05.062 27320917 PMC4927362

[27] Yang Y, Ying P, Jia Z, et al. Hyperacmotone a alleviates non-alcoholic Steatohepatitis via regulating PPARα signaling. Phytomedicine. 2025;147:157159. doi: 10.1016/j.phymed.2025.157159 40834610

[28] Zhang J, Zhang W, Yang L, et al. Phytochemical gallic acid alleviates nonalcoholic fatty liver disease via AMPK-ACC-PPARa axis through dual regulation of lipid metabolism and mitochondrial function. Phytomedicine. 2023;109:154589. doi: 10.1016/j.phymed.2022.154589 36610145

[29] Ji H, Zhang Y, Xd S, et al. Neuropeptide PACAP in mouse liver ischemia and reperfusion injury: immunomodulation by the cAMP-PKA pathway. Hepatology. 2013;57(3):1225–1237. doi: 10.1002/hep.25802 22532103 PMC3479352

[30] Schafer P, Parton A, Gandhi A, et al. Apremilast, a cAMP phosphodiesterase-4 inhibitor, demonstrates anti-inflammatory activity in vitro and in a model of psoriasis. Br J Pharmacol. 2010;159(4):842–855. doi: 10.1111/j.1476-5381.2009.00559.x 20050849 PMC2829210

[31] Parry G, Mackman N. Role of cyclic amp response element-binding protein in cyclic amp inhibition of NF-kappaB-mediated transcription. The J Immunol. 1997;159(11):5450–5456. doi: 10.4049/jimmunol.159.11.5450 9548485

[32] Oliveira-Marques V, Marinho HS, Cyrne L, et al. Role of hydrogen peroxide in NF-κB activation: from inducer to modulator. Antioxid Redox Signal. 2009;11(9):2223–2243. doi: 10.1089/ars.2009.2601 19496701

[33] Sun B, Karin M. NF-κB signaling, liver disease and hepatoprotective agents. Oncogene. 2008;27(48):6228–6244. doi: 10.1038/onc.2008.300 18931690

[34] Shi X, Li D, Deng Q, et al. Nefas activate the oxidative stress-mediated NF-κB signaling pathway to induce inflammatory response in calf hepatocytes. J Steroid Biochem Mol Biol. 2015;145:103–112. doi: 10.1016/j.jsbmb.2014.10.014 25465477

[35] Xu X, Pang Y, Fan X. Mitochondria in oxidative stress, inflammation and aging: from mechanisms to therapeutic advances. Sig Transduct Target Ther. 2025;10(1):190. doi: 10.1038/s41392-025-02253-4 PMC12159213 40500258

[36] Huang Y, Zhao C, Kong Y, et al. Elucidation of the mechanism of NEFA-induced PERK-eIf2α signaling pathway regulation of lipid metabolism in bovine hepatocytes. J Steroid Biochem Mol Biol. 2021;211:105893. doi: 10.1016/j.jsbmb.2021.105893 33819629

[37] López-Armada MJ, Caramés B, Martin M, et al. Mitochondrial activity is modulated by TNFα and IL-1β in normal human chondrocyte cells. Osteoarthr Cartil. 2006;14(10):1011–1022. doi: 10.1016/j.joca.2006.03.008 16679036

[38] Xiao X. Porcine mycoplasma pneumonia infection affects the liver transcriptome. 2025. Available from: https://dataview.ncbi.nlm.nih.gov/object/PRJNA1331453?reviewer=oohfco47kn4h4vh6aqnsm0tu08

[39] Xiao X. Integrated transcriptome and metabolome reveals Mycoplasma pneumoniae-induced lipid accumulation, inflammation, oxidative stress, and energy imbalance in the liver of pigs. Figshare. 2025. doi: 10.6084/m9.figshare.30478328 PMC13240957 42237456

